# Boosting the VZV-Specific Memory B and T Cell Response to Prevent Herpes Zoster After Kidney Transplantation

**DOI:** 10.3389/fimmu.2022.927734

**Published:** 2022-07-22

**Authors:** Marcia M. L. Kho, Willem Weimar, S. Reshwan K. Malahe, Joke M. Zuijderwijk, Ronella de Kuiper, Marieken J. Boer-Verschragen, Annemiek A. van der Eijk, Dennis A. Hesselink, Marlies E. J. Reinders, Nicole M. van Besouw

**Affiliations:** ^1^ Erasmus Medical Centre (MC) Transplant Institute, Department of Internal Medicine, University Medical Center Rotterdam, Rotterdam, Netherlands; ^2^ Department of Viroscience, Erasmus Medical Centre (MC), University Medical Centre Rotterdam, Rotterdam, Netherlands

**Keywords:** booster vaccination, herpes zoster, kidney transplantation, varicella zoster virus, humoral response, T-cell reactivity, B-cell reactivity

## Abstract

**Background:**

Solid organ transplant recipients are at high risk to develop (complicated) herpes zoster (HZ). Booster vaccination could prevent HZ. However, end-stage renal disease (ESRD) patients show poor immunological responses to vaccinations. We studied the effect of a live attenuated VZV booster vaccine on VZV-specific B and T cell memory responses in ESRD patients and healthy controls. NL28557.000.09, www.toetsingonline.nl

**Methods:**

VZV-seropositive patients, aged ≥50 years, awaiting kidney transplantation, were vaccinated with Zostavax^®^. Gender and age-matched VZV-seropositive potential living kidney donors were included as controls. VZV-specific IgG titers were measured before, at 1, 3 and 12 months post-vaccination. VZV-specific B and T cell responses before, at 3 months and 1 year after vaccination were analysed by flow-cytometry and Elispot, respectively. Occurrence of HZ was assessed at 5 years post-vaccination.

**Results:**

26 patients and 27 donors were included. Median VZV-specific IgG titers were significantly higher at all time-points post-vaccination in patients (mo 1: 3104 IU/ml [1967-3825], p<0.0001; mo 3: 2659 [1615-3156], p=0.0002; mo 12: 1988 [1104-2989], p=0.01 vs. pre: 1397 [613-2248]) and in donors (mo 1: 2981 [2126-3827], p<0.0001; mo 3: 2442 [2014-3311], p<0.0001; mo 12: 1788 [1368-2460], p=0.0005 vs. pre: 1034 [901-1744]. The patients’ IgG titers were comparable to the donors’ at all time-points. The ratio VZV-specific B cells of total IgG producing memory B cells had increased 3 months post-vaccination in patients (0.85 [0.65-1.34] vs. pre: 0.56 [0.35-0.81], p=0.003) and donors (0.85 [0.63-1.06] vs. pre: 0.53 [0.36-0.79], p<0.0001) and remained stable thereafter in donors. One year post-vaccination, the percentage of CD4+ central memory cells had increased in both patients (0.29 [0.08-0.38] vs. 0.12 [0.05-0.29], p=0.005) and donors (0.12 [0.03-0.37] vs. 0.09 [0.01-0.20], p=0.002) and CD4+ effector memory cells had increased in donors (0.07 [0.02-0.14] vs. 0.04 [0.01-0.12], p=0.007). Only 1 patient experienced HZ, which was non-complicated.

**Conclusion:**

VZV booster vaccination increases VZV-specific IgG titers and percentage VZV-specific memory T-cells for at least 1 year both in ESRD patients and healthy controls. VZV-specific memory B cells significantly increased in patients up to 3 months after vaccination. Prophylactic VZV booster vaccination prior to transplantation could reduce HZ incidence and severity after transplantation.

## Introduction

Herpes zoster (HZ) is a common complication after solid organ transplantation, incidence rates varying from 10 to 40 cases/1000 person years (1, 2) and among patients suffering from end-stage renal disease (ERSD), with hazard ratio’s from 1.4 to 3.6 compared to the general population (3, 4) The incidence of HZ in these patients is higher than the usually reported 10-12 cases/1000 person years in immunocompetent people older than 70 years (5). In addition, the disease course in immunocompromised patients is more often accompanied by severe complications, e.g. dissemination and post-herpetic neuralgia (5–7).

In the Netherlands, VZV is endemic. Most people are infected in childhood and VZV antibody prevalence is 95% in adults (8). No nationwide VZV booster vaccination program exists in the Netherlands. Previously, we showed that 96.2% of solid organ transplant candidates were VZV-seropositive (2). Prophylactic VZV vaccination in seropositive patients may boost the memory T cell and B cell repertoire and thereby reduce HZ incidence and morbidity. Unfortunately, patients with ESRD and on those on dialysis are known to build up significantly poorer antibody responses to vaccinations against influenza and hepatitis B, compared to the general population (9, 10). This is probably due to the impairing effects on the immune system of uremic toxins, malnutrition, chronic inflammation and premature thymic involution, resulting in a decreased percentage of naïve T cells and a reduction in diversity of T cell receptor repertoire in ESRD patients (11–13). After kidney transplantation, the ability to mount an adequate response to vaccination is even more impaired by immunosuppressive medication (14–17). Therefore, it makes sense to administer vaccinations prior to start of immunosuppressive medication, as recommended in major guidelines (18, 19). Only few other studies of VZV booster vaccination in patients awaiting solid organ transplantation have been performed (20–22), of which only one study reported both IgG and T cell responses (22) and none had healthy individuals as control group.

We investigated VZV-specific IgG titers, B and T cell memory responses to the live attenuated virus vaccine, Zostavax^®^, in ESRD patients awaiting kidney transplantation. Our study population was at least 50 years of age and we compared them to gender and age-matched living kidney donors.

## Materials and Methods

### Participants

The present prospective study (NL28557.000.09/MEC2009-286) was conducted between 2010 and 2015.

Patients aged ≥50 years, suffering from ESRD and awaiting renal transplantation from our outpatient clinic were enrolled. Gender- and age-matched living kidney donors were included as healthy controls. All had positive VZV-IgG titers during assessment for transplantation or donation. All participants received one dose of Zostavax^®^ (Sanofi Pasteur MSD NV, Brussels), 0.65 ml subcutaneously in an upper arm.

Gender, age, renal replacement therapy, serum anti-CMV IgG status (positive or negative) screening and cause of kidney failure (in patients) were collected from the hospital charts. These data are part of the standard medical screening for kidney transplant candidates and potential kidney donors.

Five years after vaccination, herpes zoster occurrence was assessed by reviewing the hospital electronic patient files and telephone calls to participants who did not have regular hospital visits in our center.

### Humoral Response

VZV-specific IgG antibody levels were analyzed by chemiluminescence immunoassay (Liaison^®^ XL, DiaSorin, Saluggia, Italy) before and at 1, 3 and 12 months after vaccination in all patients and donors. The cut-off for seropositivity was set at >165 mIU/ml. This automated assay for quantitative determination of VZV-specific IgG, showed good correlation with a highly sensitive VZV-IgG time-resolved fluorescence immunoassay (TRIFIA), 67% sensitivity and 100% specificity compared to TRFIA in a British population (23).

### VZV-Specific B Cell Reactivity

VZV-reactive B cell memory was determined before and at 3 and 12 months after transplantation. The VZV-specific B cell reactivity was determined by Elispot assay (24). In brief, PBMC were stimulated with B cell stimulus (U-CyTech biosciences, Utrecht, the Netherlands). After an incubation period of 5 days, the cells were transferred to a 96-well filter plates with PVDF membrane (Millipore, Darmstadt, Germany) coated with VZV antigen (Varicella Zoster grade 2 antigen; Microbix Biosystems Inc, Ontario, Canada) or anti-human IgG (U-CyTech biosciences) to determine the spontaneous frequency of IgG producing B-cells. After 5 hours of incubation, the cells were removed and biotinylated detection antibody (U-CyTech biosciences) was added. Thereafter, a streptavidin-HRP conjugate (U-CyTech biosciences) was added followed by addition of AEC (3-amino-9-ethyl-carbazole) substrate solution (U-CyTech biosciences). Spots were counted automatically by using a Bioreader 3000 Elispot reader (Biosys, GmbH, Karben, Germany). In all experiments at least 50 IgG producing cells per 1x10^4^ cells were determined. Data are presented as the ratio VZV-specific B-cells of the total IgG memory B-cells in PBMC.

### VZV-Specific T Cell Reactivity

VZV-reactive T-cell memory was determined before and at 3 and 12 months after transplantation. Mature moDCs were generated with a cocktail of cytokines as described before (25). Due to the instability of cell-free VZV in cell culture, mature moDCs were infected with VZV by co-culturing the cells with human melanoma cells (MeWo cells; American Type Culture Collection, HTB-65) infected with the vaccine Oka strain of VZV (26, 27). Mature moDCs were co-cultured with VZV-infected and mock-infected MeWo cells for 24 hours. The level of VZV infection was determined by flow cytometric analysis of the moDCs infected with VZV stained for VZV glycoprotein B (gB; Advanced Biotechnologies, Inc., Columbia, MD). Co-staining for CD86 enabled differentiation of moDCs (CD86 positive) from residual MeWo cells (CD86 negative). The level of VZV-infection was determined by subtracting the background after mock-infection from the moDCs infected with VZV. After 24 hours these moDCs were used as autologous APCs. Autologous CD3^+^ T cells were isolated from the CD14 negative fraction and incubated with moDCs infected with VZV or mock-infected moDCs for 24 hours (25) Briefly, tubes 1 and 2 contained 1x10^6^ T-cells and1x10^5^ autologous moDCs infected with VZV, and tubes 3 and 4 contained T-cells and autologous mock-infected moDCs. The cells from tubes 1 and 3 were stained with peridinin chlorophylprotein (PerCP) anti-CD4, (Becton Dickinson, Erebodegem, Belgium), allophycocyanin (APC) labeled anti-CD45RO (Becton Dickinson) and phycoerythrin (PE) labeled anti-CCR7 (R&D Systems Europe Ltd, Abingdon, UK). The cells from tubes 2 and 4 were stained with PerCP-labeled anti-CD8 (Becton Dickinson), APC-labeled anti-CD45RO (BectonDickinson) and PE-labeled anti-CCR7 (R&D Systems) (**Figure S1**). Thereafter, the cells from all tubes were fixed and permeabilized followed by incubation with anti-human IFN-γ. The VZV-reactive T cells were determined by counting the number of IFN-γ producing CD3^+^CD4^+^ and CD3^+^CD8^+^ T cells stimulated with autologous moDCs infected with VZV, minus the respective values obtained upon stimulation with autologous mock-infected moDCs, on the fluorescence-activated cell sorter (FACS Canto-II, Becton Dickinson). The VZV-reactive T cells are expressed as percentage of the total number of reactive T cells or (central or effector) memory cells.

### Statistical Analysis

Statistical analyses were performed in SPSS, version 25, 2017 and GraphPad Prism 9.1.2, 2021.

Patient and donor categorical variables were compared using Fisher’s exact and Pearson chi square tests and continuous variables with Mann-Whitney test. VZV-IgG titers and VZV-specific T and B cell responses were compared between patients and donors with Mann-Whitney U test and within patient and donor groups using Wilcoxon signed rank test. Data are presented as median with interquartile range. VZV-IgG geometric mean fold rise (GMFR) was calculated for each time point after vaccination: geometric mean titer (GMT) at that time point divided by GMT pre-vaccination.

## Results

### Participants

**Figure 1:**
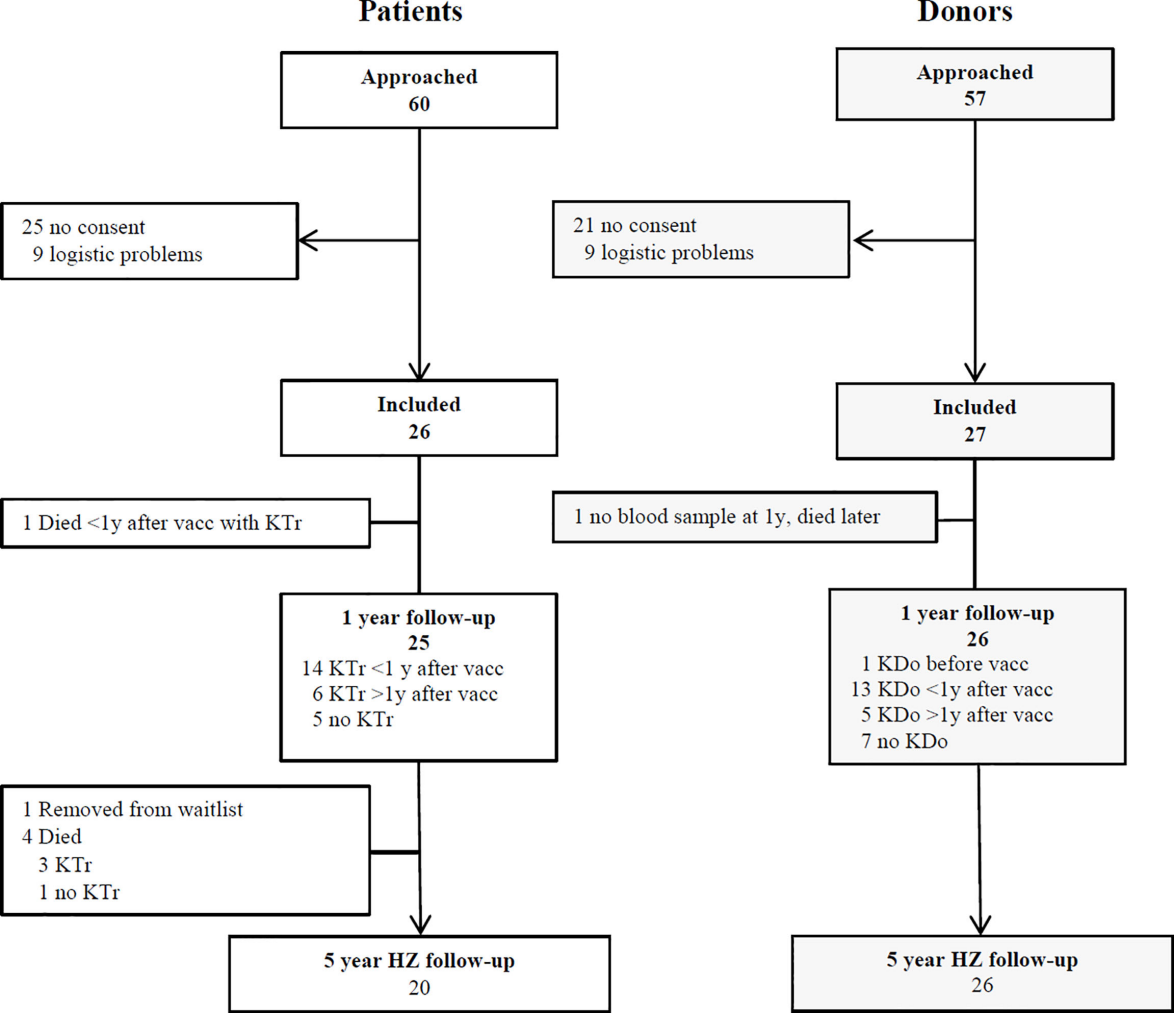
Participants enrolment and follow-up. KTr= kidney transplant. KDo= kidney donation. HZ= herpes zoster. Vacc= vaccination. Eligible, potential participants were approached at the outpatient clinic and by telephone call from the investigators. They received both oral and written information. Some participants could not be included due to a tight schedule of transplantation and donation and/or temporary unavailability of the vaccine.

A total of 26 patients and 27 donors was included (**Figure 1**). The characteristics of the patients and donors are listed in **Table 1**. Gender, age and CMV serostatus were comparable between patients and donors. The follow-up of patients and donors is described in **Table 2**.

**Table 1:** Patient and donor characteristics at vaccination.

	Patients	Donors	p
**Number**	26	27	
**Gender (M/F)**	14/12	12/15	0.59
**Age^a^ (years)**	64 (50-77)	62 (52-74)	0.44
**RRT**			
** No/HD/PD**	17/6/3		
**CMV-IgG**			
** pos/neg/unknown**	19/7/0	14/9/4	0.54
**Renal disease**			
** Hypertension** ** Diabetes Mellitus** ** Polycystic** ** Glomerular disease^b^** ** Other^c^**	8 6 5 4 3		

M: male, F: female

a : median (range)

RRT: renal replacement therapy. HD: hemodialysis. PD: peritoneal dialysis

CMV-IgG: anti-cytomegalovirus Immunoglobulin G

b : 1x glomerulonephritis eci, 2x IgA nephropathy, 1x Alport’s disease

c : 1x acute tubular necrosis due to sepsis, 1x unknown, 1x nephrolithiasis due to cystinuria

**Table 2:** Patient and donor follow-up.

	Kidney Transplant Recipients			Kidney Donors		
	Yes	No	p	Yes	No	p
**Number (%)**	21 (81)	5 (19)		19 (70)	8 (30)	
**<1 year post vaccination, n (%)**	15 (58)			13 (48)		
**>1 year post vaccination, n (%)**	6 (23)			5 (19)		
**pre-vaccination, n (%)**	0			1 (4)		
**Age at vaccination** ^**a**^	63 (50-74)	71 (61-77)	0.41	64 (52-73)	62 (51-66)	0.41
**Donor type, n (%)**						
**Living** **Deceased**	14 (67) 7 (33)					
**Time to transplantation/donation** **(months post vaccination)** ^**a**^	13.5 (1-67)			4.9 (**-**2-47)		
**RRT**						
**No/HD/PD**	13/5/3	4/1/0				
**Herpes Zoster, n (%)**	1 (4.8)	0		0	0	
**Death** ^**b**^, **n (%)**	4 (19)	1 (20)		0	1 (12.5)	
**Time to death (months)** ^**a**^						
**since vaccination** **since transplantation/donation**	61 (6-91) 36 (0.3-80)	18.7 -			13.6 -	

RRT= renal replacement therapy. HD= hemodialysis. PD= peritoneal dialysis.

a : median + range

b : Four transplant recipients: 1 heart failure, 1 malignancy: lung carcinoma, 1 infection: cellulitis + sepsis, 1 unknown. One patient without transplant: heart failure. One donor: retroperitoneal sarcoma

#### Patients

Seventeen patients were waiting for a pre-emptive living or deceased donor kidney transplantation, whereas six patients were on hemodialysis and 3 on peritoneal dialysis (**Table 1**). Twenty-one patients received a kidney transplant, 15 within 1 year after vaccination and 6 more than 1 year (range 24 – 67 months) after vaccination. Five patients did not receive a kidney transplant during the study period: 3 were waiting but not on dialysis, 1 died due to heart failure and 1 was removed from the waitlist due to severe iliac artery atherosclerosis. Four patients died after transplantation (**Table 2**).

The characteristics of the patients who received a transplant after vaccination are described in **Table 3**. Five patients experienced a rejection episode within the first year (0.1 to 8 months) after transplantation, varying from 3 to 15 months after vaccination (**Table 3**). Their anti-HLA antibody level, expressed as panel reactive antibody (PRA) did not increase after vaccination. Two of these 5 rejections occurred in ABO-incompatible transplants.

**Table 3:** Kidney transplant recipient characteristics.

	No rejection	Rejection	p
**Number**	16	5	
**Age (years)** ^**a**^	64 (50-74)	62 (54-70)	0.40
**RRT**			
** No/HD/PD**	11/4/1	2/1/2	
**Donor type, n (%)**			
** Living** ** Deceased**	10 (62.5) 6 (37.5)	4 (80) 1 (20)	0.35
**Time to KTr (months after vaccination)** ^**a**^	7 (1-67)	5 (2-12)	
**Time to rejection (months)** ^**a**^			
** since vaccination** ** since transplantation**		5.8 (3-15) 0.2 (0.2-8)	
**Herpes Zoster, n (%)**	0	1 (20)	
**Time to Herpes Zoster (months)** ^**a**^			
** since vaccination** ** since transplantation**		15.6 10.8	
**PRA** ^**b**^			
** before vaccination** ** at transplantation**	8.7 (0-77) n=15 0.0 (0-0) n=11	2.6 (0-13) n=5 2.0 (0-8) n=4	0.30 0.27
**ABO-incompatible kidney transplantation** **n (%)**	1 (6)	2 (40)	
**Induction therapy, n (%)**			
** Basiliximab** ** Rituximab** ^**c**^ ** Alemtuzumab**	15 (94) 0 1 (6)	3 (60) 2 (40) 0	
**Maintenance immunosuppression, n (%)**			
** Tac + MMF** ** Tac + MMF + pred** ** Other**	11 (69) 1 (6) 4 (25) ^d^	1 (20) 2 (40) 2 (40)^e^	

KTr= kidney transplantation. RRT= renal replacement therapy. HD= hemodialysis. PD= peritoneal dialysis. Tac= tacrolimus. MMF= mycophenolate mofetil. Pred= prednisolone

a : median + range

b : PRA= panel reactive antigen as percentage, mean + range, n= patients with available PRA

c : because of ABO-incompatible kidney transplantation

d : 2 Tac monotherapy, 1 everolimus + MMF + pred, 1 Tac + pred

e : Tac + pred

#### Donors

Nineteen donors donated a kidney, 13 within 1 year after vaccination and 5 more than 1 year (range 15 – 47 months) after vaccination. One donor donated 2 months before vaccination. One potential donor died due to a malignancy almost 14 months after vaccination (**Table 2**). Prior to her disease, she and her recipient had been removed from the transplant program because of severe iliac artery atherosclerosis in the recipient.

### VZV-Specific IgG

**Figure 2:**
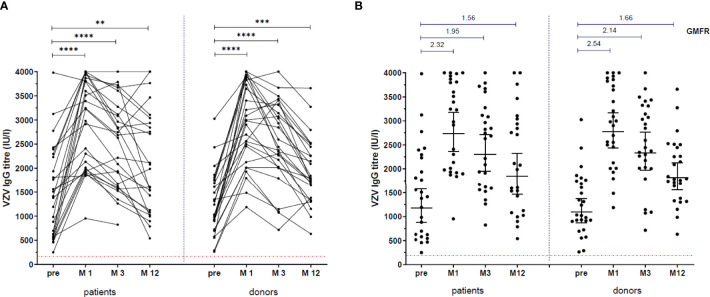
VZV-specific IgG response. VZV-specific IgG titres, before vaccination and at 1 month (M1), 3 months (M3) and 12 months (M12) after vaccination. **(A)** **: p=0.005, ***: p=0.0002, ****: p<0.0001. Patients compared to donors: pre: p=0.67, M1: p=0.94, M3: p=0.79, M12: p=0.84. **(B)** Lines in scatter plots indicate Geometric Mean with 95% confidence interval. Above the scatter plots: Geometric Mean Fold Rise (GMFR) between pre and M1, M3 and M12.

25/26 patients and 26/27 donors reached the 12 month time point. VZV-specific IgG titers were significantly higher at all time-points after vaccination in patients (M1: 3104 IU/ml [1967-3825], p<0.0001; M3: 2659 [1615-3156], p=0.0002; M12: 1988 [1104-2989], p=0.01 vs. pre: 1397 [613-2248]) and in donors (M1: 2981 [2126-3827], p<0.0001; M3: 2442 [2014-3311], p<0.0001; M12: 1788 [1368-2460], p=0.0005 vs. pre: 1034 [901-1744] (**Figure 2A**). The patients’ titers were comparable to the donors’ titers at all time points: pre: p=0.64, mo 1: p=0.94, mo 3: p=0.79, mo 12: p=0.84. GMFR was also comparable between patients and donors at all time points (**Figure 2B**).

**Figure 3:**
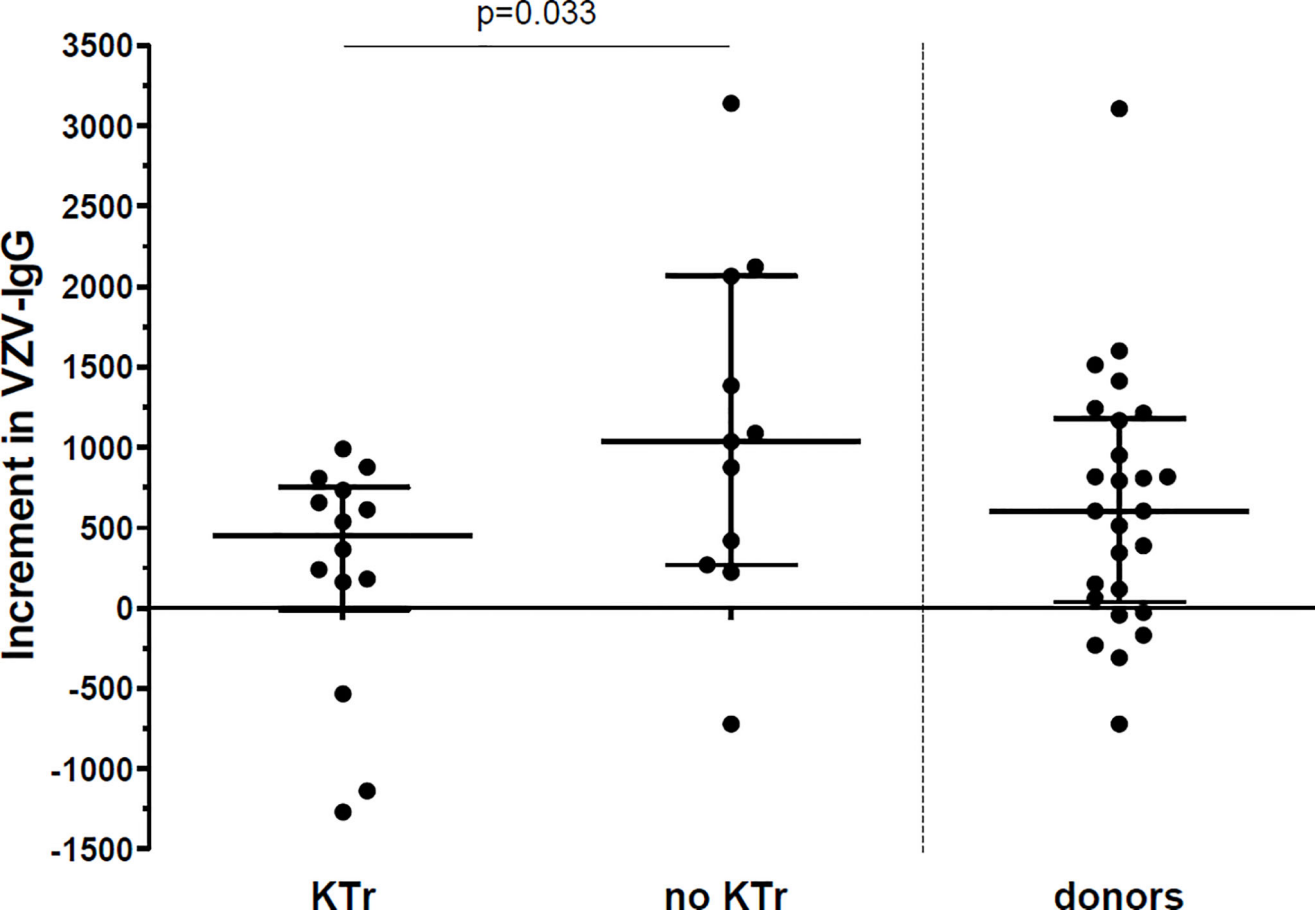
Increment in VZV-IgG titres between vaccination and 12 months after vaccination. Increment in VZV-IgG titres in 14 patients who received a kidney transplant (KTr) within 1 year after vaccination compared to 11 patients who were still on the waitlist (no KTr) at 1 year after vaccination and to 26 donors. Lines indicate median with interquartile range.

The patients who did not receive a kidney transplant within 1 year post vaccination had a greater increment in IgG titers between pre-vaccination and month 12 (median 1035 [268-2063]), compared to the patients who received a transplant within the first year (450 [-13-751], p=0.033) (**Figure 3**).

We found no difference in VZV IgG titers at any time point between patients who were on dialysis at vaccination and patients who were not (**Table S1**).

Also, no difference was found between VZV IgG titers at any time point and CMV serostatus at vaccination in patients (**Table S2**) and donors (**Table S3**).

### VZV-Specific B Cell Response

**Figure 4:**
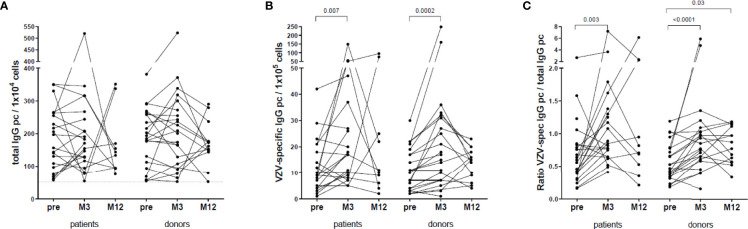
VZV-specific B cell response. **(A)** Total IgG producing B cells per 10^4^ cells. **(B)** VZV-specific IgG producing B cells per 10^5^ cells. **(C)** Ratio between VZV-specific IgG producing B cells and total IgG producing B cells.

VZV-reactive B cell memory was determined pre-vaccination in 22 patients and 22 donors, at 3 months in 21 patients and 21 donors and at 12 months in 10 patients and 14 donors. Total numbers of IgG producing cells were similar between patients and donors, within the patient group and within the donor group (**Figure 4A**). The number of VZV-specific IgG producing memory B cells increased significantly within the first 3 months post-vaccination in both patients and donors (**Figure 4B**). Between patients and donors, the numbers of VZV-specific IgG producing B cells were similar. The ratio VZV-specific B cells of the total IgG producing memory B cells in PBMC had also increased significantly at month 3 in both patients (0.85 [0.65-1.34] vs. pre: 0.56 [0.35-0.81], p=0.003) and donors (0.85 [0.63-1.06] vs. pre: 0.53 [0.36-0.79], p<0.0001) (**Figure 4C**). In donors this ratio remained significantly higher at month 12 compared to pre-vaccination (**Figure 4C**).

VZV-specific B cell data were only available in 6 patients who received a kidney transplant within 1 year after vaccination and in 4 patients who did not. Due to these low numbers of patients, statistical analysis was not performed.

We found no difference in the ratio VZV-specific of total IgG producing B cells at any time point between patients who were on dialysis (n=7) at vaccination and patients who were not (n=15) (**Table S1**).

No difference was found between the ratio VZV-specific of total IgG producing B cells at any time point and CMV serostatus at vaccination in patients (n=22: 5 IgG neg, 17 IgG pos) (**Table S2**) and donors (n=19: 8 IgG neg, 11 IgG pos) (**Table S3**).

### VZV-Specific T Cell Response

VZV-reactive T cell memory was determined in 18 patients and 22 donors at all time points. We compared the VZV-reactive memory cells at 3 and 12 months after vaccination with before vaccination.

**Figure 5:**
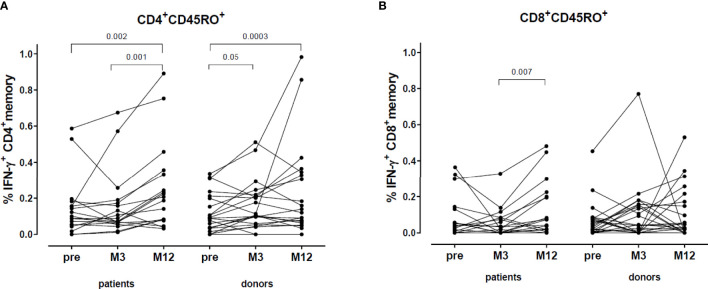
VZV-reactive T cell response: CD4^+^ and CD8^+^ memory. **(A)** Percentage VZV-specific IFN-γ producing CD4^+^ memory cells. **(B)** Percentage VZV-specific IFN-γ producing CD8^+^ memory cells.

The percentage of VZV-reactive CD4^+^ memory cells (defined as CD4^+^CD45RO^+^IFN- γ^+^) significantly increased in both donors (M3 and M12 compared to pre-vaccination) and patients (M12 compared to pre-vaccination and M12 compared to M3) (**Figure 5A**). No relevant difference was found in the VZV-reactive CD8^+^ memory response (defined as CD8^+^CD45RO^+^IFN- γ^+^) (**Figure 5B**).

**Figure 6:**
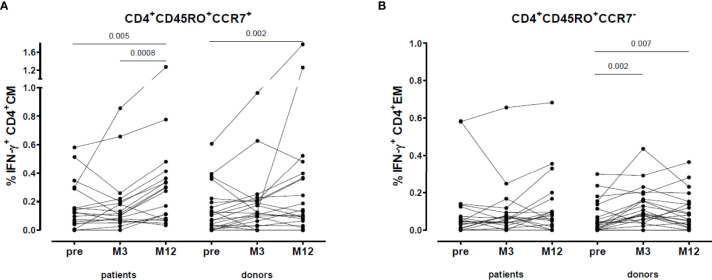
VZV-reactive T cell response: CD4^+^ central and effector memory. **(A)** Percentage VZV-specific IFN-γ producing CD4^+^ central memory cells. **(B)** Percentage VZV-specific IFN-γ producing CD4^+^ effector memory cells.

When we divided the VZV-reactive CD4^+^ memory cells into CD4^+^ central memory (CM, CD4^+^CD45RO^+^CCR7^+^) and effector memory (EM, CD4^+^CD45RO^+^CCR7^-^) cells, we found that in both patients (0.29 [0.08-0.38] vs. 0.12 [0.05-0.29], p=0.005) and donors (0.12 [0.03-0.37] vs. 0.09 [0.01-0.20], p=0.002) the percentage of VZV-reactive CD4^+^ CM cells increased after vaccination and were still higher at one year after vaccination (**Figure 6A**). However, the VZV-reactive CD4^+^ EM cells only increased in donors (0.07 [0.02-0.14] vs. 0.04 [0.01-0.12], p=0.007) (**Figure 6B**).

VZV-specific T cell memory data were available in 11 patients who received a kidney transplant within 1 year after vaccination and in 8 patients who did not. No difference was found at month 12 in percentage of VZV- reactive CD4^+^ (total, CM and EM) nor in VZV- reactive CD8^+^ memory cells between patients with and without a kidney transplant and between both patient groups and donors (**Table S4**).

We found no difference in VZV-reactive CD4^+^ or in VZV-reactive CD8^+^ memory cells at any time point between patients who were on dialysis at vaccination (n=6) and patients who were not (n=12) (**Table S1**).

No difference was found between VZV-reactive CD4^+^ or VZV-reactive CD8^+^ memory cells at any time point and CMV serostatus at vaccination in patients (n=18: 4 IgG neg, 14 IgG pos) (**Table S2**) and donors (n=18: 7 IgG neg, 11 IgG pos) (**Table S3**).

### Adverse Events After Vaccination

A 68-year-old female donor developed an itching rash on face and breast (no blisters), a feeling of malaise and slightly elevated temperature one day post-vaccination. She spontaneously recovered in 4 days after vaccination. None of the other participants reported adverse events.

### Herpes Zoster After Vaccination

One patient suffered from a mild HZ, localized just below her left breast. It occurred 16 months after vaccination, 11 months after transplantation and 9 months after anti-rejection treatment (methylprednisolone and IVIG). She was treated with oral valaciclovir for 9 days. She recovered completely within one month.

## Discussion

Natural immunity against VZV is maintained during (subclinical) virus reactivation and re-exposure to the virus but declines as the immune system ages (28). From hepatitis B vaccination in dialysis patients and SARS-CoV2 vaccination in kidney transplant patients, it is known that higher and repeated dosing (boostering) can improve antibody and cellular response against the virus (11, 29–31). As herpes zoster incidence and severity are high in solid organ transplant recipients (1, 2, 4), it is important to investigate whether booster vaccination can decrease HZ incidence. Vaccine efficacy can be assessed by monitoring both the humoral and cellular VZV response after booster vaccination. To effectively control VZV reactivation, cell mediated immunity is necessary and the magnitude of the cellular response correlates better with HZ severity than IgG titers (28, 32–34). Weinberg et al. reported that that in elderly people, a higher cell mediated immune response (measured in VZV responder cell frequency and IFN-γ Elispot) correlated with reduced HZ morbidity, whereas VZV antibody titers did not (32). In a nonhuman primate model with simian varicella virus infection, it was shown that depletion of CD4^+^, and not CD8^+^, T cells resulted in significantly higher viral loads and disseminated varicella, while the absence of B cells did not alter disease severity (33). Given the helper functions of CD4^+^ T cells, their absence would lead to delay and reduction of VZV-specific antibody and CD8^+^ T cell responses (28). CD8^+^ T cell response to VZV has been studied less extensively than CD4^+^ T cell responses.

To our knowledge, this is the first study comparing three parameters reflecting the immune response to VZV booster vaccination (IgG titers, B cell and T cell memory) in ESRD patients and healthy controls (kidney donors), all above 50 years of age. We found that at 3 months after Zostavax^®^, VZV-specific IgG titers and B cell memory had equally increased in ESRD patients compared to controls. At 1 year after vaccination, VZV-specific IgG titers remained significantly high in both groups, but the ratio VZV-specific memory B cells of the total IgG producing memory B cells had declined in patients. The percentage of VZV-reactive CD4^+^ T cells and central memory CD4^+^ cells were significantly increased at 1 year in both patients and controls. The percentage of VZV-reactive CD4^+^ effector memory cells were only significantly increased in controls.

From the few studies describing immune responses to VZV booster vaccination in patients awaiting solid organ transplantation (20–22), only one study concerned ESRD patients. Miller et al. compared VZV antibody titers after Zostavax^®^ (n=26) and after placebo vaccine (n=8). Geometric mean titer was significantly higher in the Zostavax group only at 5 weeks after vaccination, but not at 12 months (20). Comparing the study of Miller et al. with our study, a higher percentage of patients (69%) was on dialysis at time of vaccination and only 46% received a kidney transplant thereafter, while in our study these percentages were 35% and 81% (58% within 1 year post vaccination), respectively. In the transplant recipients, Miller et al. demonstrated a more pronounced decline in antibodies, while we found comparable levels of VZV IgG antibodies between transplant recipients and healthy donors (**Figures 2A, B**). In our study, the patients who received a kidney transplant within one year after vaccination, had lower VZV IgG titers compared to those still awaiting transplantation. Indeed, several reports demonstrated that especially mycophenolic acid impairs B cell numbers and production of IgG (14, 15). However, we found no difference in percentage VZV-specific T cells between patients who received their transplant within one year after vaccination and those who did not. Tacrolimus, the most used calcineurin inhibitor in our transplant recipients, inhibits T cell activation, including CD4^+^ helper function, and T cell proliferation. Despite this suppressive effect, it has been shown that VZV specific CD4^+^ and CD8^+^ memory T cells did significantly increase after a herpes zoster episode in lung transplant patients (24). VZV-reactive memory CD4^+^, but not CD8^+^ T cells also significantly increased upon *in vitro* stimulation by VZV infected dendritic cells in kidney transplant patients (25). Recently, Wang et al. also found that in patients vaccinated prior to lung transplantation, VZV stimulated IFN-γ producing cells decreased shortly after lung transplantation, but had increased again at 6 months or longer after transplantation (22). In patients with latent VZV infection, circulating VZV-specific CD4^+^ memory T cells are long-lived, and these cells have skin-homing and tissue residing ability (28). This could be one mechanism by which these cells escape the effect of immunosuppressive drugs.

We did not find any difference in immune responses to the booster vaccination between our dialysis and ESRD patients. A possible explanation could be that duration of dialysis before vaccination was limited (median 12 months, range 5-48). Tseng et al. reported that a lower HZ incidence in vaccinated ESRD patients is most prominent when vaccination was performed within 2 years of dialysis initiation (35).

Only one patient experienced a mild and self-limiting rash after vaccination. In 5 of 21 transplant recipients an acute rejection episode was observed. Acute rejection incidence in the vaccinated transplant recipients was comparable to the general acute rejection incidence in our center in the same time period (36). Therefore, we conclude that vaccination was safe and did not induce graft rejection. With a follow-up of 5 years, only 1 patient suffered from herpes zoster, with mild symptoms no complications and full recovery. None of the controls had herpes zoster.

However, the present study was not designed to detect significant differences in herpes zoster incidence. Furthermore, only patients who were not using immunosuppressive medication could be included, because the Zostavax vaccine contains live attenuated virus. Although there are many reports on the safety of zoster vaccines (34), caution is advised when live attenuated virus vaccination is considered in immunocompromised patients. A literature review from Price et al. reported three cases of fatal zoster vaccine infections. Of these patients, one was taking prednisone 10 mg/day, methotrexate and hydroxychloroquine and two patients had a hematologic malignancy without use of immunosuppressive medication at least 6 months before administration of the zoster vaccine (37). Since 2018, a recombinant subunit adjuvanted vaccine (Shingrix^®^) was also approved in Europe to prevent HZ. In the Netherlands, Shingrix^®^ was only available from June 2020. As this vaccine does not contain live attenuated virus, it may possibly be given to patients using immunosuppressive drugs. Efficacy and safety have been reported in a phase 3 study with kidney transplant recipients (38). However, regardless of the vaccine type, performing vaccination in patients *before* they receive a kidney transplant is an obvious strategy. Also SARS-CoV-2 vaccination studies have shown that vaccine immunogenicity in patients with chronic kidney disease and even on dialysis is better compared to in patients with a kidney transplant (39, 40).

In conclusion, our study showed that boosting the immune system of ESRD patients ≥50 years old with Zostavax^®^ does significantly increase VZV-specific IgG titers and CD4^+^ memory cells, even to comparable levels as in healthy controls of the same age. The responses persisted for at least one year after vaccination, despite the introduction of immunosuppressive medication after kidney transplantation. Given the high herpes zoster incidence after solid organ transplantation, it seems justified to perform booster vaccination in transplant candidates.

## Data Availability Statement

The raw data supporting the conclusions of this article will be made available by the authors, without undue reservation.

## Ethics Statement

The studies involving human participants were reviewed and approved by the Central Committee on Research Involving Human Subjects in The Netherlands (NL28557.000.09) and the Medical Ethics Committee of the Erasmus MC (MEC2009-286).

## Author Contributions

MK and NV designed the research, collected, analyzed and interpreted the data and prepared the manuscript. WW designed the research. JZ and RD performed the laboratory tests. MB-V administered the vaccinations and participated in the clinical follow-up of the participants. SM, AV, DH and MR participated in interpretation and preparation of the manuscript.

## Funding

This study was supported in part by a research grant from the Investigator-Sponsored Studies Program of Sanofi Pasteur MSD. Sanofi Pasteur MSD supplied the Zostavax^®^ vaccines for this study grant number: ZOS-2010-002.

## Conflict of Interest

Author MK received a honorarium for participation in a scientific advisory board from Takeda. Author DH has received lecture fees and consulting fees from Astellas Pharma, Chiesi Pharma, Medincell, Novartis Pharma, Sangamo Therapeutics and Vifor Pharma. He has received grant support from Astellas Pharma, Bristol-Myers Squibb and Chiesi Pharma [paid to his institution]. Author DA Hesselink does not have employment or stock ownership at any of these companies, and neither does he have patents or patent applications.

The remaining authors declare that the research was conducted in the absence of any commercial or financial relationships that could be construed as a potential conflict of interest.

## Publisher’s Note

All claims expressed in this article are solely those of the authors and do not necessarily represent those of their affiliated organizations, or those of the publisher, the editors and the reviewers. Any product that may be evaluated in this article, or claim that may be made by its manufacturer, is not guaranteed or endorsed by the publisher.

## Supplementary Material

Supplementary Figure 1 Representative example of the gate setting. From the lymphocyte gate, the CD3^+^ cells were selected. Thereafter, the CD3^+^ CD4^+^ and CD8^+^ cells were gated. From the CD4^+^ and CD8^+^ cells the naïve, central memory and effector memory cells were selected.

## Abbreviations

ABO-I, ABO-blood type incompatible; CMV, cytomegalovirus; ESRD, end stage renal disease; GMFR, geometric mean fold rise; GMT, geometric mean titer; HLA, human leucocyte antigen; HZ, herpes zoster; IFN-γ, interferon-γ; IgG, immunoglobulin G; IVIG, intravenous immunoglobulin; PBMC, peripheral blood mononuclear cell; PRA, panel reactive antigen; SARS-CoV-2, severe acute respiratory syndrome coronavirus 2; SOT, solid organ transplantation; VZV, varicella zoster virus.
