# Supplementary figures and images for: Boosting the VZV-Specific Memory B and T Cell Response to Prevent Herpes Zoster After Kidney Transplantation
Source: Front Immunol. 2022 Jul 22;13:927734. doi: 10.3389/fimmu.2022.927734 (PMC9352887; doi:10.3389/fimmu.2022.927734)

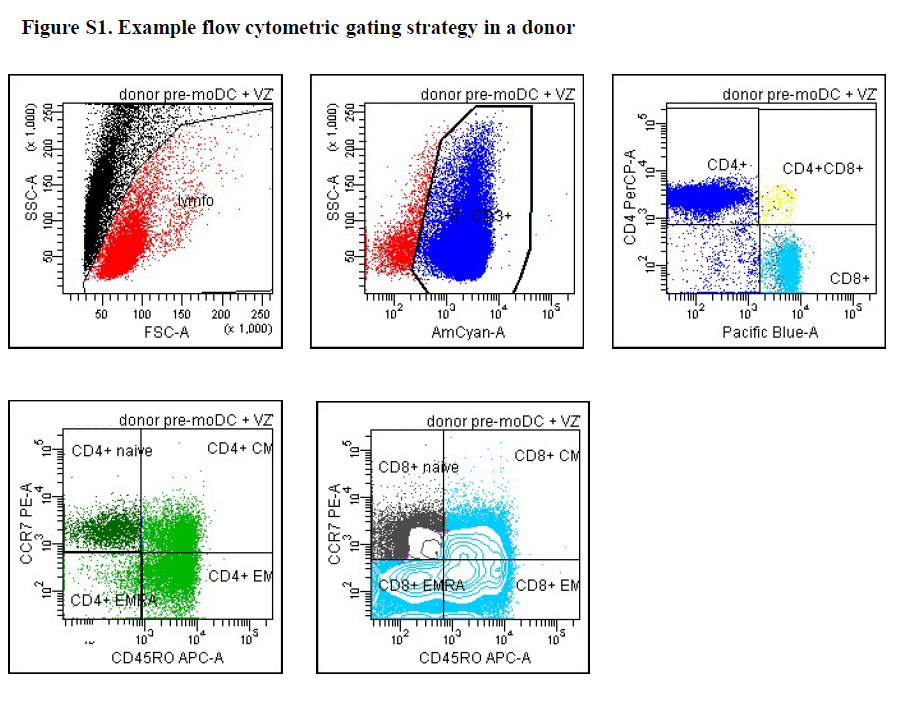

Supplement: Supplementary Figure 1 — Representative example of the gate setting. From the lymphocyte gate, the CD3+ cells were selected. Thereafter, the CD3+ CD4+ and CD8+ cells were gated. From the CD4+ and CD8+ cells the naïve, central memory and effector memory cells were selected. [file Image_1.tif]
