# Supplementary material for: Boosting the VZV-Specific Memory B and T Cell Response to Prevent Herpes Zoster After Kidney Transplantation
Source: Front Immunol. 2022 Jul 22;13:927734. doi: 10.3389/fimmu.2022.927734 (PMC9352887; doi:10.3389/fimmu.2022.927734)
Supplement: Supplementary file 2 [file DataSheet_1.pdf]

Table S1

			CD4 TM pre	CD4 TM M3	CD4 TM M12	CD4 CM pre	CD4 CM M3	CD4 CM M12	CD4 EM pre	CD4 EM M3	CD4 EM M12
Recipients	Dialysis no	median	0,105	0,120	0,210	0,131	0,157	0,318	0,057	0,074	0,091
		25% percentile	0,046	0,066	0,083	0,043	0,081	0,089	0,016	0,041	0,050
		75% percentile	0,156	0,240	0,433	0,263	0,249	0,464	0,139	0,156	0,299
	Dialysis yes	median	0,095	0,067	0,145	0,112	0,088	0,192	0,040	0,042	0,030
		25% percentile	0,054	0,046	0,043	0,055	0,054	0,045	0,006	0,000	0,000
		75% percentile	0,186	0,106	0,229	0,304	0,138	0,309	0,063	0,068	0,097
	Dialysis no vs. yes	P-value	0,892	0,180	0,213	0,964	0,250	0,151	0,291	0,102	0,083

			CD8 TM pre	CD8 TM M3	CD8 TM M12	CD8 CM pre	CD8 CM M3	CD8 CM M12	CD8 EM pre	CD8 EM M3	CD8 EM M12
Recipients	Dialysis no	median	0,041	0,012	0,078	0,010	0,009	0,041	0,049	0,062	0,103
		25% percentile	0,002	0,000	0,017	0,000	0,000	0,000	0,014	0,000	0,007
		75% percentile	0,141	0,084	0,219	0,146	0,106	0,157	0,284	0,116	0,208
	Dialysis yes	median	0,039	0,035	0,040	0,017	0,027	0,001	0,061	0,039	0,015
		25% percentile	0,000	0,014	0,004	0,000	0,005	0,000	0,014	0,006	0,000
		75% percentile	0,114	0,138	0,177	0,139	0,166	0,279	0,063	0,075	0,088
	Dialysis no vs. yes	P-value	0,682	0,553	0,682	0,964	0,437	0,820	0,553	0,553	0,125

			B cell pre	B cell M3	B cell M12		VZV-IgG T=0 vacc	VZV-IgG T=1 mo	VZV-IgG T=3 mo	VZV-IgG T=12 mo
Recipients	Dialysis no	median	0,420	0,780	0,710		1380,0	2979,0	2630,0	1988,0
		25% percentile	0,290	0,620	0,530		626,8	2073,5	1647,5	1278,0
		75% percentile	0,790	1,300	2,260		2084,5	3839,5	3440,0	3073,0
	Dialysis yes	median	0,600	1,070	0,950		1559,0	3391,0	2844,0	1805,5
		25% percentile	0,460	0,800	0,210		563,1	1869,5	1469,5	1039,0
		75% percentile	1,230	2,133	6,130		2536,0	3791,5	3100,0	2881,0
	Dialysis no vs. yes	P-value	0,185	0,235	0,833		0,792	0,597	0,916	0,588

Table S2

			CD4 TM pre	CD4 TM M3	CD4 TM M12	CD4 CM pre	CD4 CM M3	CD4 CM M12	CD4 EM pre	CD4 EM M3	CD4 EM M12
Recipients	CMV IgG neg	median	0,086	0,062	0,065	0,100	0,067	0,081	0,059	0,070	0,055
		25% percentile	0,018	0,023	0,036	0,018	0,016	0,038	0,020	0,049	0,013
		75% percentile	0,142	0,160	0,270	0,147	0,183	0,338	0,120	0,089	0,087
	CMV IgG pos	median	0,106	0,098	0,216	0,131	0,124	0,300	0,046	0,053	0,091
		25% percentile	0,051	0,066	0,084	0,046	0,086	0,104	0,006	0,021	0,027
		75% percentile	0,186	0,193	0,382	0,311	0,216	0,393	0,129	0,131	0,234
	CMV neg vs. pos	P-value	0,574	0,277	0,158	0,442	0,192	0,192	0,789	0,645	0,277

			CD8 TM pre	CD8 TM M3	CD8 TM M12	CD8 CM pre	CD8 CM M3	CD8 CM M12	CD8 EM pre	CD8 EM M3	CD8 EM M12
Recipients	CMV IgG neg	median	0,054	0,046	0,149	0,109	0,139	0,343	0,062	0,058	0,070
		25% percentile	0,013	0,008	0,050	0,009	0,002	0,019	0,016	0,013	0,017
		75% percentile	0,238	0,261	0,417	0,380	0,470	0,615	0,099	0,201	0,119
	CMV IgG pos	median	0,030	0,020	0,030	0,000	0,018	0,000	0,043	0,040	0,025
		25% percentile	0,000	0,000	0,004	0,000	0,000	0,000	0,015	0,000	0,002
		75% percentile	0,134	0,084	0,196	0,048	0,038	0,146	0,151	0,104	0,207
	CMV neg vs. pos	P-value	0,721	0,574	0,158	0,158	0,382	0,061	1,000	0,798	0,798

			B cell pre	B cell M3	B cell M12	VZV-IgG T=0 vacc	VZV-IgG T=1 mo	VZV-IgG T=3 mo	VZV-IgG T=12 mo
Recipients	CMV IgG neg	median	0,600	1,250	2,340	882,5	3228,0	2630,0	2078,0
		25% percentile	0,435	0,995	0,690	470,0	1934,0	1913,0	1081,0
		75% percentile	0,925	4,410	6,130	1937,0	4000,0	3116,0	2713,0
	CMV IgG pos	median	0,460	0,775	0,710	1510,0	2979,0	2687,0	1797,0
		25% percentile	0,340	0,625	0,350	624,2	1978,0	1561,0	1100,8
		75% percentile	0,840	1,245	0,950	2393,0	3810,0	3274,0	3056,5
	CMV neg vs. pos	P-value	0,704	0,062	0,183	0,334	0,651	0,611	0,929

Table S3

			CD4 TM pre	CD4 TM M3	CD4 TM M12	CD4 CM pre	CD4 CM M3	CD4 CM M12	CD4 EM pre	CD4 EM M3	CD4 EM M12
Donors	CMV IgG neg	median	0,034	0,054	0,048	0,035	0,033	0,031	0,035	0,108	0,082
		25% percentile	0,000	0,000	0,000	0,000	0,000	0,000	0,000	0,000	0,000
		75% percentile	0,153	0,219	0,305	0,159	0,198	0,360	0,138	0,231	0,153
	CMV IgG pos	median	0,106	0,176	0,159	0,105	0,173	0,142	0,044	0,088	0,120
		25% percentile	0,034	0,059	0,078	0,034	0,064	0,092	0,014	0,039	0,027
		75% percentile	0,309	0,293	0,425	0,375	0,206	0,523	0,155	0,194	0,198
	CMV neg vs. pos	P-value	0,151	0,179	0,069	0,246	0,246	0,126	0,536	0,860	0,536

			CD8 TM pre	CD8 TM M3	CD8 TM M12	CD8 CM pre	CD8 CM M3	CD8 CM M12	CD8 EM pre	CD8 EM M3	CD8 EM M12
Donors	CMV IgG neg	median	0,063	0,040	0,047	0,104	0,024	0,065	0,056	0,070	0,043
		25% percentile	0,022	0,000	0,000	0,044	0,000	0,001	0,000	0,012	0,000
		75% percentile	0,138	0,106	0,216	0,169	0,105	0,270	0,177	0,168	0,245
	CMV IgG pos	median	0,019	0,026	0,019	0,028	0,002	0,029	0,006	0,029	0,025
		25% percentile	0,000	0,000	0,000	0,000	0,000	0,000	0,000	0,000	0,000
		75% percentile	0,072	0,155	0,057	0,070	0,217	0,342	0,039	0,070	0,099
	CMV neg vs. pos	P-value	0,211	0,659	0,596	0,126	0,930	0,659	0,375	0,285	0,536

			B cell pre	B cell M3	B cell M12	VZV-IgG T=0 vacc	VZV-IgG T=1 mo	VZV-IgG T=3 mo	VZV-IgG T=12 mo
Donors	CMV IgG neg	median	0,460	0,820	0,775	1239,0	2499,0	2175,0	1381,0
		25% percentile	0,325	0,650	0,568	825,3	1747,0	1466,5	1308,5
		75% percentile	0,875	1,018	1,123	1648,0	3787,5	3087,5	1811,5
	CMV IgG pos	median	0,470	0,870	0,870	1146,7	2989,5	2393,0	2091,0
		25% percentile	0,320	0,570	0,558	659,3	2097,5	2012,0	1703,0
		75% percentile	0,720	1,155	1,150	1748,8	3866,5	3162,8	2516,0
		P-value	1,000	0,762	0,818	0,877	0,369	0,600	0,051

Table S4

			CD4 TM M12	CD4 CM M12	CD4 EM M12	CD8 TM M12
**Recipients**	**KT<1y: no**	**median**	0,164	0,253	0,056	0,020
		**25% percentile**	0,054	0,069	0,022	0,004
		**75% percentile**	0,271	0,673	0,268	0,073
	**KT<1y: yes**	**median**	0,224	0,298	0,096	0,075
		**25% percentile**	0,083	0,111	0,000	0,005
		**75% percentile**	0,458	0,413	0,168	0,299
**Donors**		**median**	0,106	0,117	0,070	0,038
		**25% percentile**	0,050	0,028	0,024	0,000
		**75% percentile**	0,332	0,374	0,145	0,183
	**KT<1y no vs. yes**	**P-value**	0,351	0,904	0,887	0,168
	**KT<1y no vs. Donors**	**P-value**	0,872	0,467	0,989	0,357
	**KT<1y yes vs. Donors**	**P-value**	0,233	0,221	0,873	0,327
